# Supplementary material for: Genome-Wide Analysis of Cyclic Nucleotide-Gated Channel Genes Related to Pollen Development in Rice
Source: Plants (Basel). 2022 Nov 17;11(22):3145. doi: 10.3390/plants11223145 (PMC9692566; doi:10.3390/plants11223145)
Supplement: Supplementary file 1 [file plants-11-03145-s001.zip › plants-1930103-supplementary.pdf]

Additional data

Table S1. Os*CNGC gene* isogene-specific primers for qPCR and pGA3574 cloning.

Genes	Sense Primers	Antisense Primers
**qPCR**		
OsCNGC4	5'-GCGGCAGTACATCAACGAGAC-3'	5'-AACGTCGCCAGGAATATCGA-3'
OsCNGC5	5'-CAGTACCGGTGGCTGGAGAC-3'	5'-TCCGTGCACAGGCTGGGCTT-3'
OsCNGC8	5'-ATCCGCTACCTCAAGAACGA -3'	5'-TCGAGAGCGTGTTGTTGGTG-3'
**p3574 cloning (For Protoplast system)**		
OsCNGC4	5'-CTGCAGCCCGGGGGATCCATGTCGTACGCGTCCGGTG-3'	5'-CTTGCTCACCATACTAGTGTAATCGGCGCCGAAATCAGGCTCG-3'
OsCNGC5	5'-CTGCAGCCCGGGGGATCCATGTTTGGCAGCTGCGGC-3'	5'-CTTGCTCACCATACTAGTGTACTCCATGGTCTGGAAGTCAGGC-3'
OsCNGC8	5'-CTGCAGCCCGGGGGATCCATGGACATGCAGATCGGC-3'	5'-CTTGCTCACCATACTAGTCAGCACGTCGTCGGAGCTGAGG-3'
**Yeast vector cloning**		
CNGC4-(pGADT7)	5'-GCCATGGAGGCCAGTGAATTCGAGATGCGGGTGAAGCGG-3'	5'-AGCTCGAGCTCGATGGATCCTCAGTAATCGGCGCCGAAATCA-3'
CNGC4-(pGBKT7)	5'-GCCATGGAGGCCAGTGAATTCGAGATGCGGCTGCGGCGC-3'	5'-CGCTGCAGGTCGACGGATCCTCAGTAATCGGCGCCGAAATCA-3'
CNGC5-(pGADT7)	5'-GCCATGGAGGCCAGTGAATTCGAATGGCGGGTGAAGCGG-3'	5'-AGCTCGAGCTCGATGGATCCTCAGTACTCCATGGTCTGGAAG-3'
CNGC5-(pGBKT7)	5'-ATGGCCATGGAGGCCGAATTCGAGATGCGG GTGAAGCGG-3'	5'-CGCTGCAGGTCGACGGATCCTCAGTACTCCATGGTCTGGAAG-3'
CNGC8-(pGADT7)	5'-ATGGCCATGGAGGCCGAATTCGAGATGCGGCTGCGGCGC-3'	5'-AGCTCGAGCTCGATGGATCCTCACAGCACGTCGTCGGAGC-3'
CNGC8-(pGBKT7)	5'-ATGGCCATGGAGGCCGAATTCGAATGGCGGGTGAAGCGG-3'	5'-CGCTGCAGGTCGACGGATCCTCACAGCACGTCGTCGGAGC-3'

Table S2. Accession numbers of *CNGC* genes

Gene	Locus	Gene	Locus
OsCNGC1	LOC_Os02g15580	AtCNGC1	Loc_At5g53130
OsCNGC2	LOC_Os06g33570	AtCNGC2	Loc_At5g15410
OsCNGC3	LOC_Os06g33610	AtCNGC3	Loc_At2g46430
OsCNGC4	LOC_Os03g44440	AtCNGC4	Loc_At5g54250
OsCNGC5	LOC_Os12g28260	AtCNGC5	Loc_At5g57940
OsCNGC6	LOC_Os04g55080	AtCNGC6	Loc_At2g23980
OsCNGC7	LOC_Os02g41710	AtCNGC7	Loc_At1g15990
OsCNGC8	LOC_Os12g06570	AtCNGC8	Loc_At1g19780
OsCNGC9	LOC_Os09g38580	AtCNGC9	Loc_At4g30560
OsCNGC10	LOC_Os02g54760	AtCNGC10	Loc_At1g01340
OsCNGC11	LOC_Os06g08850	AtCNGC11	Loc_At2g46440
OsCNGC12	LOC_Os02g53340	AtCNGC12	Loc_At2g46450
OsCNGC13	LOC_Os06g10580	AtCNGC13	Loc_At4g01010
OsCNGC14	LOC_Os03g55100	AtCNGC14	Loc_At2g24610
OsCNGC15	LOC_Os01g57370	AtCNGC15	Loc_At2g28260
OsCNGC16	LOC_Os05g42250	AtCNGC16	Loc_At3g48010
		AtCNGC17	Loc_At4g30360
		AtCNGC18	Loc_At5g14870
		AtCNGC19	Loc_At3g17690
		AtCNGC20	Loc_At3g17700

Table S3. Analysis of pollen-preferred CREs in Os*CNGCs*

	Pollen-preferred CREs		
	**POLLEN1LELAT52 (AGAAA)**	**GTGANTG10 (GTGA)**	**PB CORE (CACC)**
OsCNGC1	12	9	8
OsCNGC2	2	13	22
OsCNGC3	11	7	8
OsCNGC4	2	14	18
OsCNGC5	5	10	24
OsCNGC6	9	18	13
OsCNGC7	19	11	10
OsCNGC8	5	13	23
OsCNGC9	16	9	8
OsCNGC10	14	11	21
OsCNGC11	8	13	9
OsCNGC12	11	10	17
OsCNGC13	8	15	15
OsCNGC14	12	12	19
OsCNGC15	8	16	20
OsCNGC16	9	18	15

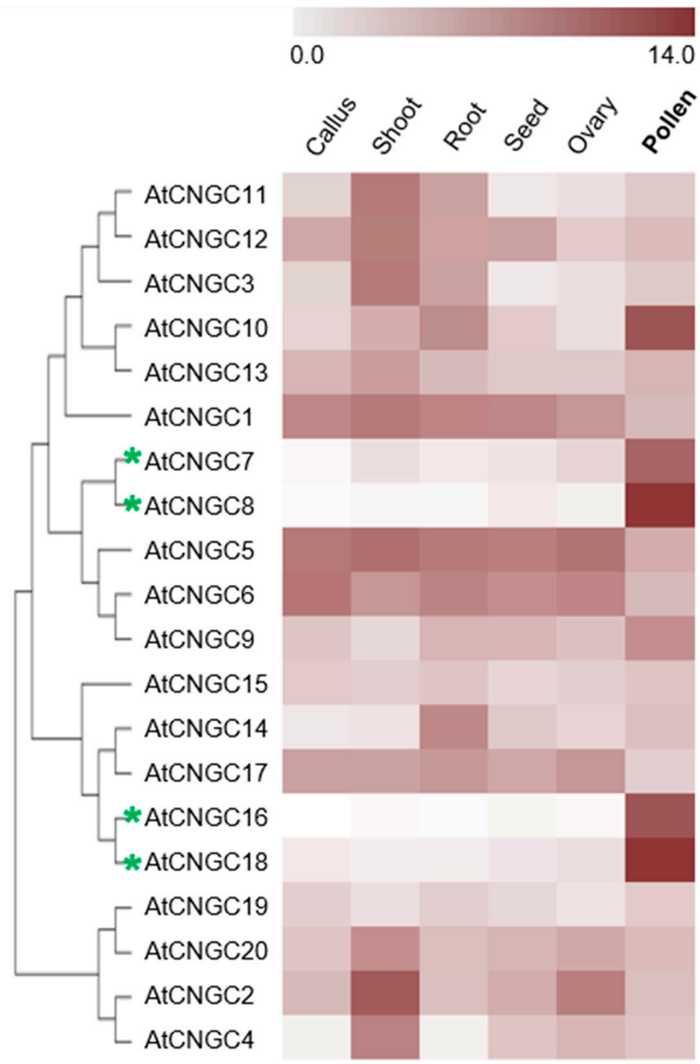

Figure S1. Pollen specific expression patterns of 20 CNGC genes in Arabidopsis thaliana from the public database (http://bar.utoronto.ca/efp/cgi-bin/efpWeb.cgi). Data from gene expression map of Arabidopsis development based on microarray and RNA-seq. These expression patterns suggest the role of the *CNGC* genes in the rice, which has a close relationship with the above pollen preferred Arabidopsis genes indicated by green asterisks (AtCNGC7, AtCNGC8, AtCNGC16, AtCNGC18).

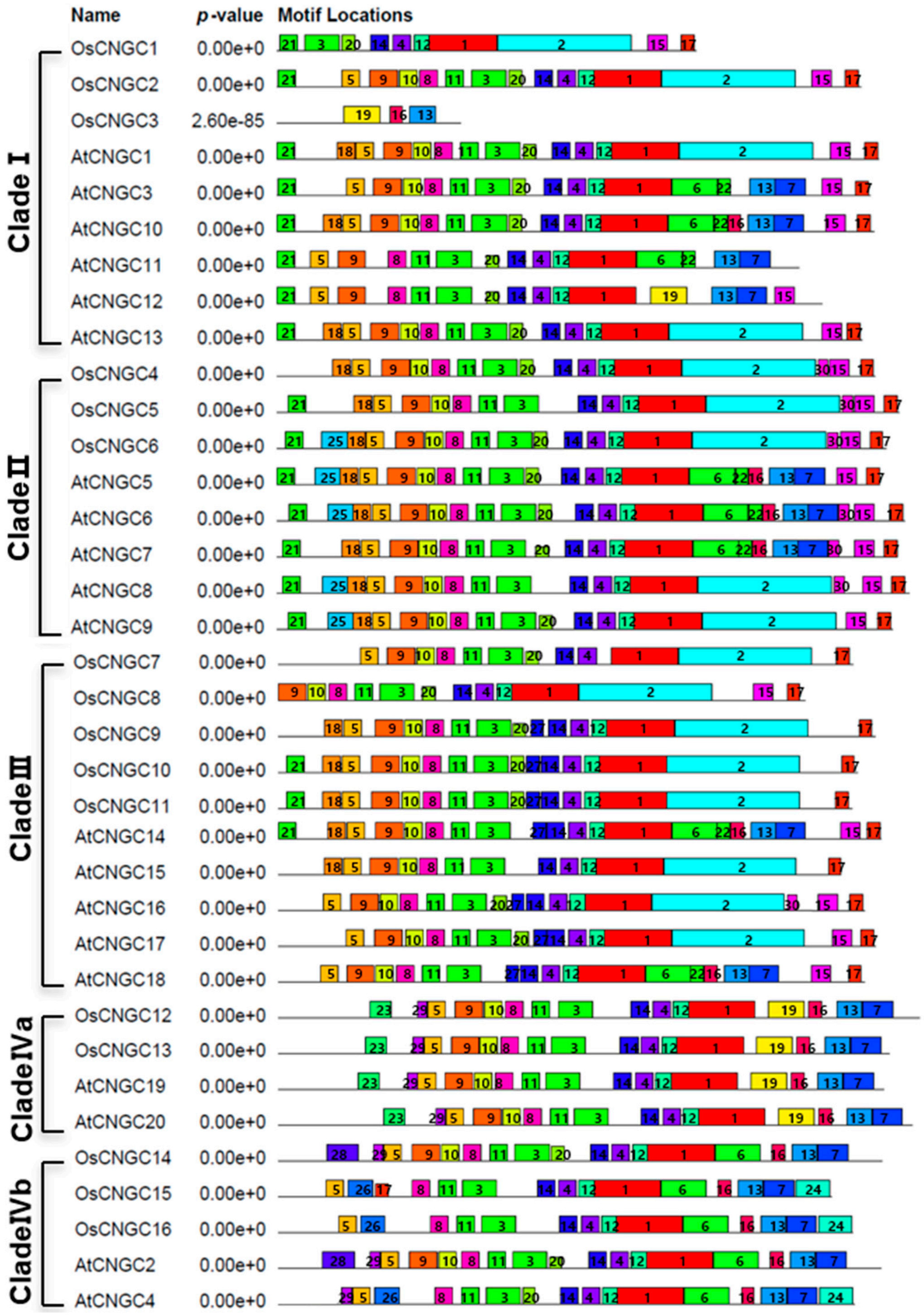
Name
p-value
Motif Locations
Clade I
OsCNGC1 0.00e+0
OsCNGC2 0.00e+0
OsCNGC3 2.60e-85
AtCNGC1 0.00e+0
AtCNGC3 0.00e+0
AtCNGC10 0.00e+0
AtCNGC11 0.00e+0
AtCNGC12 0.00e+0
AtCNGC13 0.00e+0
Clade II
OsCNGC4 0.00e+0
OsCNGC5 0.00e+0
OsCNGC6 0.00e+0
AtCNGC5 0.00e+0
AtCNGC6 0.00e+0
AtCNGC7 0.00e+0
AtCNGC8 0.00e+0
AtCNGC9 0.00e+0
Clade III
OsCNGC7 0.00e+0
OsCNGC8 0.00e+0
OsCNGC9 0.00e+0
OsCNGC10 0.00e+0
OsCNGC11 0.00e+0
AtCNGC14 0.00e+0
AtCNGC15 0.00e+0
AtCNGC16 0.00e+0
AtCNGC17 0.00e+0
AtCNGC18 0.00e+0
Clade IVa
OsCNGC12 0.00e+0
OsCNGC13 0.00e+0
AtCNGC19 0.00e+0
AtCNGC20 0.00e+0
Clade IVb
OsCNGC14 0.00e+0
OsCNGC15 0.00e+0
OsCNGC16 0.00e+0
AtCNGC2 0.00e+0
AtCNGC4 0.00e+0

Motif	Symbol	Motif Consensus
1.		LIGNMQTYLQSLTVRLEEMRVKRRDTEQWMSHRQLPEELRERVRRYEQYKWLATRGVDEENLLQNLPKDLRRDIKRHLCL
2.		LVRRVPLFENMDEQLLDAICERLKPSLYTEGTYIVREGDPVBEMLFIIRGKLESSTTBGGRTGFFNSILLKPGDFCGEELLTWA LDPKSASNLPSSTRTVKALTEVEAFALRAEDLKFVASQFRRLHSKQLQHTFRFYSHQWRTWAACFIQAAWRRYKR
3.		GVVAETAWAGAAYNLLLYMLASHVVGALWYLLSIZRQDTCW
4.		FYCLWWGLQNLSTLGQNLETS
5.		WNKIFLJSCIVALFVDPLFFY
6.		LVRRVPLFQHMDDLLLDAICDRLKPLLFTEGEYIVREGDPVQEMLFIIRGRLZS
7.		LHSKKVQHTFRYYSPQWRTWAACFIQAAWRRYKRRK
8.		FIIDLLAVLPLPQIVIWLVIP
9.		IDRKLAITVTVLRTVIDLFYLLHILLQFRTAYI
10.		SSRVFGRGELVIDPKKIAKRY
11.		KNALLLIVLFQYIPRIYRIYP
12.		EVJFAIFIAISGLVLFAL
13.		STRTVRALTEVEAFALSAEDLKFVASQFRR
14.		NNTPFNFGIYTDALSSGVVSS
15.		SSGSSLSLGATJLASRFAANALR
16.		EGDFCGEELLTWALD
17.		LPLLQKPPEPDFSAD
18.		VFPEDLEVSEKKIFDPQDKFL
19.		DAICDRLKQRLYIAGSTILHQGGPVEKMVFIVRGKLESISADG
20.		CKNNPGCNLRFLYCG
21.		MNGRREKFVRFDDEDSRGSSS
22.		TTDGGRSGFFNSVLL
23.		SGPLGMCDDPDCVTCPSYYKRKRAF
24.		SLSFIRPRRPLSRCSSLGEEKLRLYTAILTSPKPNPDDDD
25.		SSRSFKKGVRKGSEGLKSIGRSJGLGVSRA
26.		PLMCVFLDGWLAAAVTALRCMVDAMHAWN
27.		QNWANVTAVFSNCDA
28.		ECYACTQPGVPAFHSTSCDQVHAPEWDADAGSSLVPIQ
29.		GPIMBPHSKRVQ
30.		AELRRKEEEEEE

Figure S2. MEME domain analysis and schematic diagram for main motif structures of CNGC genes in rice and *Arabidopsis.* Each motif is represented by a number in colored box.

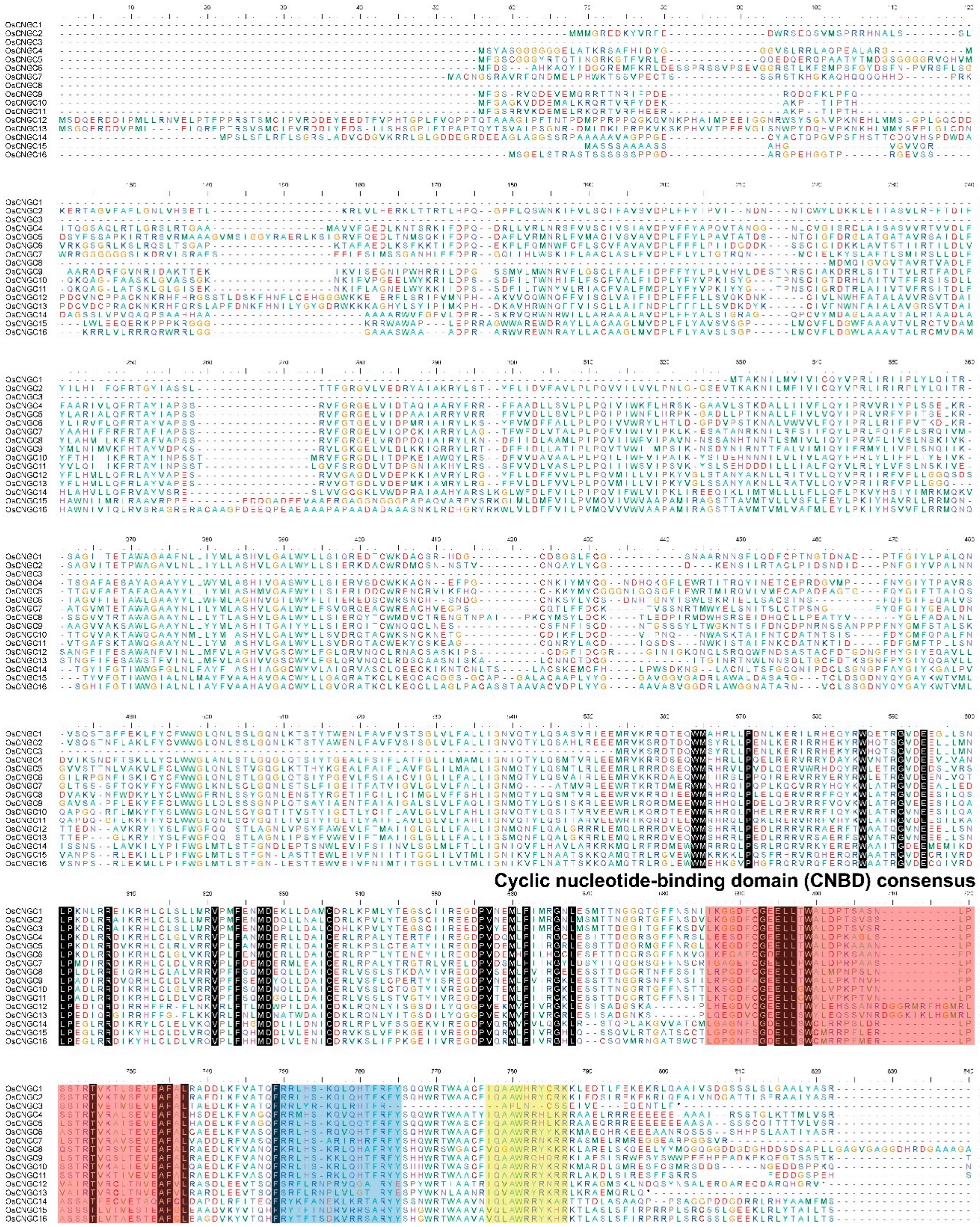

Figure S3. Protein sequence alignment domain analysis and conserved phosphorylated amino acid of protein sequences encoded by Os*CNGC* genes. 16 OsCNGC protein sequences were collected and aligned. CNGC signature motif LI-X(2)-[GS]-X-[FV]-X-G-[1]-ELL-X-W-X(12,22)-SA-X(2)-T-X(7)-[EQ]-AF-XL is well conserved in C-terminal site; the red boxes. Additionally, the yellow boxes indcate CT-IQ motif that is an extremely basic unit of about 23 amino acids and their conserved core usually fits the consensus A-x(3)-I-Q-x(2)-F-R-x(4)-K-K.. The blue box indicates CaMBD and all three domains serve as a binding site for 2nd messengers in various signaling pathways.

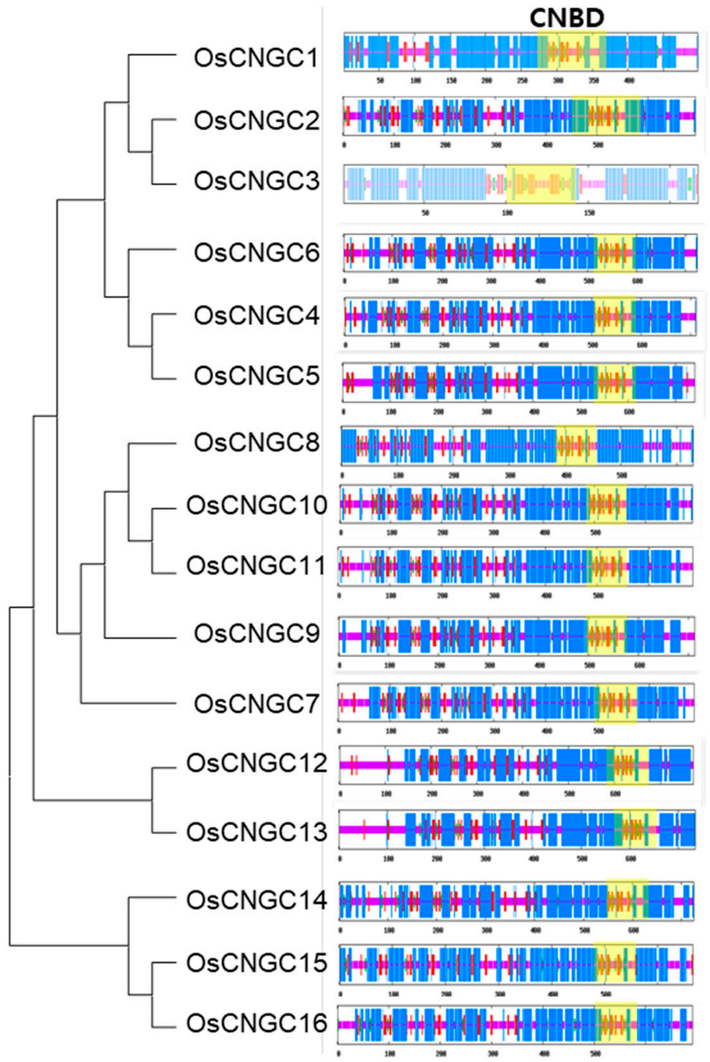

Figure S4. Secondary structure of CNGC proteins using the deduced amino acid sequences. Blue lines indicate alpha helices, purple lines indicate random coils, red lines denote extended strands, and green lines represent beta turns. The yellow boxes indicate CNBD that is representative characteristic among CNGC genes.

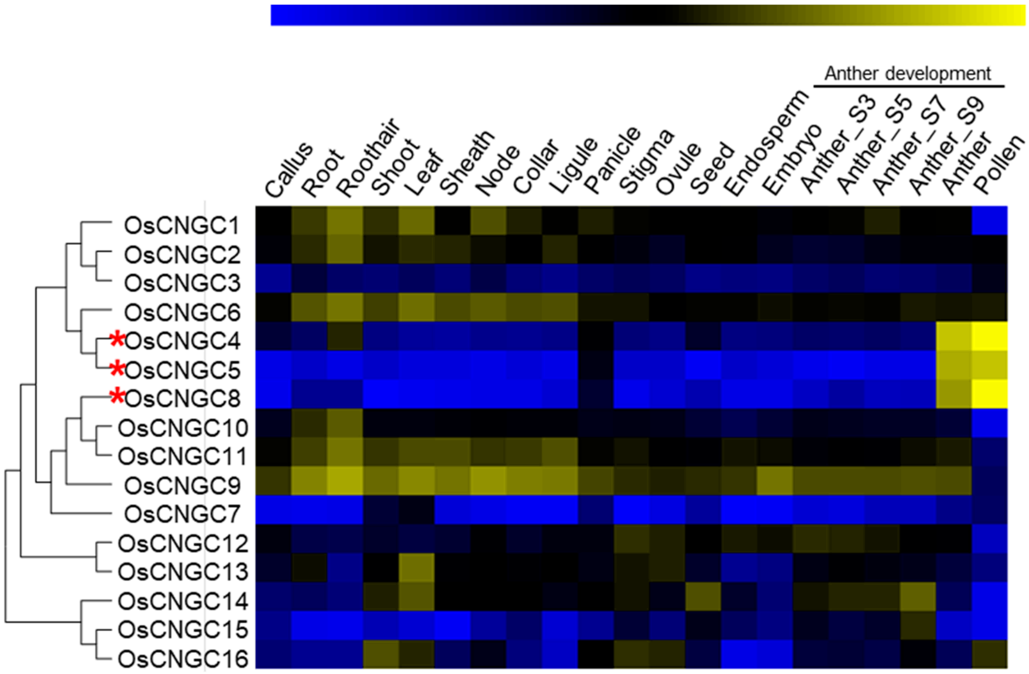

Figure S5. Heatmap expression and phylogenetic analysis of 16 CNGC genes in 17 tissues/organs, and exceptionally anther tissues include at a variety of developmental stages. Yellow color in the heatmap indicates a high level of expression, whereas dark blue indicates low expression. Numeric values indicate an average of the normalized log2 intensity value of the microarray data. Pollen-preferentially expressed OsCNGC genes are indicated by red asterisks.
